# Cochlear Implant Activation in the Immediate Postoperative Period in the Operating Room

**DOI:** 10.1055/s-0043-1776722

**Published:** 2024-01-24

**Authors:** Gislaine Richter Minhoto Wiemes, Nicole Richter Minhoto Wiemes, Bettina Carvalho, Rogerio Hamerschmidt

**Affiliations:** 1 Department of Speach Therapy, Hospital Paranaense de Otorrinolaringologia, Curitiba, PR, Brazil; 2 Department of ENT, Universidade Federal do Paraná, Curitiba, PR, Brazil

**Keywords:** cochlear implantation, bilateral hearing loss, telemetry, impedance

## Abstract

**Introduction** Cochlear implant (CI) activation usually takes place at ∼ 30 days postoperative (PO). In our service, CI surgery is performed with local anesthesia and sedation, so activation is possible with the patient's cooperation, immediately after the CI surgery, still in the operating room (OR).

**Objective** The objective of the present study was to provide the patient with hearing experience with the CI and to assess auditory perception immediately after surgery while still in the OR, as well as to compare impedance telemetry (IT), neural response telemetry (NRT), and comfort (C) level at two moments: in the OR and at the definitive activation, ∼ 30 days PO.

**Methods** Nine adult patients (12 ears) with acquired (postlingual) deafness were included. Auditory perception was evaluated through the Ling Six Sound Check, musical instruments, and clapping, presented in two different programming maps, elaborated using t-NRT, and comparing IT, NRT, and C level between the two moments.

**Results** We observed that while still in the OR, the patient can already present auditory detection and recognition responses. The values of IT, NRT threshold (t-NRT), and C on both dates differed, with statistical significance.

**Conclusion** We concluded that it is possible to provide the patient with an auditory experience with the CI immediately after surgery, and that the auditory experience and the values of electrode IT, NRT, and C vary significantly between the two moments.

## Introduction

Cochlear implants (CIs) are well established as a successful tool for providing individuals with severe-to-profound bilateral hearing loss the access to sound and speech. It consists of an internal and one external unit, which has a speech processor that is normally activated in the speech therapist's office, ∼ 30 to 40 days after surgery, which is the amount of time necessary for adequate wound healing. Its parameters are adjusted often using data obtained during surgery: stimulus current level, speed, and pulse width. As the auditory perception elicited by the CI depends on the amount of electrical current that passes through the system, and the amount of current needed to elicit auditory sensation is different for each individual and for each stimulation channel, the electrical stimulation parameters must be individually adjusted to suit the needs of each patient. This is a process called *mapping* , which is performed by the speech therapist through a software connected to the speech processor. The more accurate the mapping, the greater the potential for achieving adequate speech perception. Mapping can be performed subjectively, through conditioning techniques and behavioral observation (clinical assessment), or objectively, through exams. 1

We know that adults give better feedback at the time of activation, and, in our service, we perform CI surgery with local anesthesia and sedation, 2 so it is possible to activate the CI with patient cooperation, immediately after surgery, in adults, still in the operating room (OR).

The objective of the present work was to carry out activation immediately after surgery while still in the OR, thus providing the patient with an auditory experience with the CI; additionally, we wanted to determine the dynamic field (T and C levels) and to perform impedance telemetry (IT) and neural response telemetry (NRT), which is based on the measure of the electric compound action potential (ECAP) thresholds, using two different programming maps (maps 1 and 2) and to compare them at the moment of surgery and at the definitive activation, 30 days later.

## Materials and Methods

This was a prospective, analytical, longitudinal study, approved by the institutional review board under number 12855619.9.0000.5529. It included 9 adult patients (6 unilateral and 3 bilateral cases, 12 ears total) with acquired (postlingual) deafness, who underwent CI surgery under local anesthesia and sedation according to our standard protocol 2 and who consented to the CI activation in the operating room (OR).

Patients either already had a CI in one ear and underwent sequential surgery or were using hearing aids and underwent CI surgery.

First, in the OR, IT was performed to assess the integrity and functionality of the electrodes. Impedances were measured on the 22 electrodes in monopolar MP1, monopolar MP2, monopolar MP1 + 2, and common ground (CG) modes. Values were considered normal when between 1.5 and 20 kΩ in MP1, MP2, and MP1 + 2 modes and between 0.7 and 20 kΩ in CG mode. Only electrodes that presented impedance within the normal limits, according to software standards, were used for recording NRT. Then, measurements of intraoperative t-NRT (NRT threshold) were performed in all electrodes, and we used the response in at least 5 electrodes for analysis, dividing the cochlea into 4 regions: electrodes 01 to 05, 06, to 10, 11 to 15, and 16 to 22. The current level (CL) in each electrode initiated at 150 CL, with an interval of 6 CL between one stimulus and the other, up to the maximum stimulation of 255 CL, or until reaching t-NRT. Interpulse interval was fixed at 500 µs, stimulation speed at 80 Hz in series of 25 µs per phase.

We used Cochlear Corporation Custom Sound EP software (Cochlear Limited, Sydney, Australia) to obtain objective measurements of IT and NRT, and Custom Sound to assemble maps and perform activation following surgery. With the Nucleus 5 - CP 810 processor (Cochlear Limited), 2 maps were created with stimulation speed of 900 Hz, 8 maxima, volume 6, sensitivity 12, with different levels of T and C:

- Map 1: Created with C levels by subtracting 10 current units from tNRT;
- Map 2: Created with C levels equal to tNRT.

And T levels were estimated at 40 current units below C level.

**Fig. 1:**
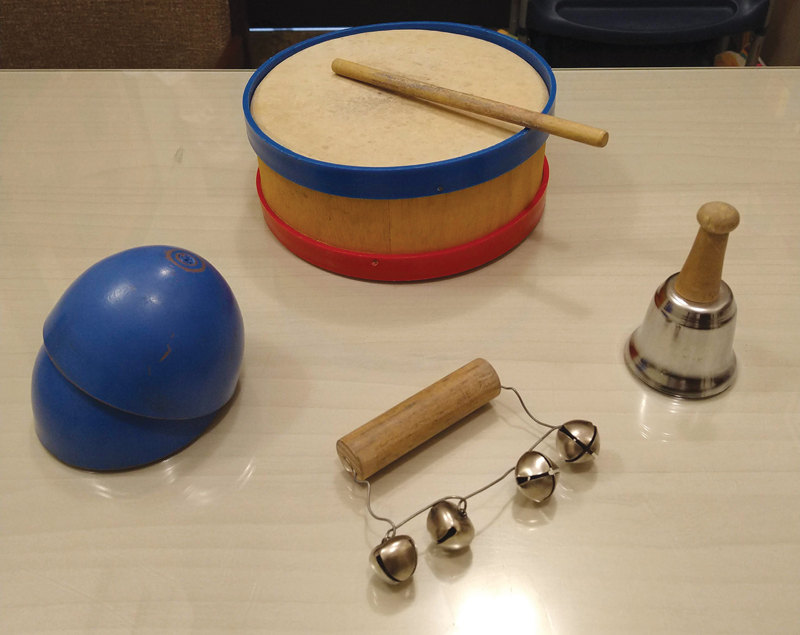
Instruments used in the activation.

Afterwards, each patient was evaluated in the OR, with either map, by:

- Ling sounds (/a/, /i/, /u/, /m/, /s/, /sh/);
- Instrumental sounds: bell rattle (2 KHz to 6 KHz), coconut shells (600 Hz to 3 KHz), bell (4 KHz–8 KHz), and drum (250–600 Hz) (Russo and Santos, 1994); ( Fig. 1 )
- Claps (one or two claps)

Presentations were performed in a closed set, with the patient being informed about which sounds would be presented: Ling sounds, musical instruments, or clapping. All were performed ∼ 60 cm from the speech processor. The patient was still lying on the surgical table. Responses were observed by the same speech therapist and noted. The analysis of the auditory perception responses, immediately after surgery, in the two different maps tested, was done by observational analysis. The behavioral tests were evaluated based on the auditory skills: detection, discrimination, and recognition.

The second assessment was done on the day of the definite activation, 30 days postoperatively, with IT and NRT.

Results were described as mean, standard deviation (SD), median, minimum, and maximum. To compare NRT and IT between the two evaluation moments, the Student *t* -test was used for paired samples. The normality condition was evaluated by the Shapiro-Wilk test. Values of *p* < 0.05 were considered of statistical significance. Data were analyzed using the computer program Stata/SE v.14.1. (StataCorpLP, College Station, TX, USA. Detection, recognition, and non-detection to sound were considered for the observational analysis of responses to Ling sounds, instrumental, and clapping sounds.

## Results

The analysis performed was based on data from 12 ears of 9 patients (6 unilateral and 3 bilateral cases). The CI electrode bundle CI24RE (CA) (Cochlear Limited) was used for 10 ears, and CI422 for 2 ears. Based on the presented tNRT, maps 1 and 2 were tested. We based the analysis on the fact that sound detection precedes auditory recognition, so all percentage calculations were based on the total detection and recognition being 100%, and from this we calculated the percentage of recognition.

For the detection, non-detection and recognition of Ling sounds in the 12 ears, we found that with Map 1: the 12 ears detected the phoneme /a/, but only 3 ears recognized it, the 12 ears detected /i/, but only 2 recognized it, 11 detected /u/, 3 recognized it, 11 detected /m/, 12 detected /sh/ and one recognized it, 12 detected /s/. With map 2, for the phoneme /a/ 12 detected and 5 recognized it, for /i/ 12 detected and 2 recognized it, for /u/ 12 detected and 6 recognized it, for /m/ 12 detected and 2 recognized it, for /sh/ 12 detected and 4 recognized it, and for /s/ 12 detected and 1 recognized it. Based on these data we can say that we obtained 12.85% of recognition of LING sounds for map 1 and 27.77% with map 2.

For the detection, non-detection, and auditory recognition of musical instruments with map 1, there was detection of the bell rattle, coconut shells, bell, and drum for the 12 ears, 3 of which recognized the bell and 3 recognized the drum. As for map 2, there was detection of the bell rattle for 11 ears, for the other instruments there was detection with the 12 ears, and 2 ears recognized the bell, and 3 the drum. Therefore, it was possible to observe 25% of recognition of the bell and drum for map 1 and 16.66% of recognition for the bell and 25% for the drum with map 2.

For the detection, non-detection, and recognition of one or two claps, we verified that claps were possible to be detected and recognized. With map 1 we found 41.66% of recognition for one or two claps and with map 2 the recognition was 33.33%. With both map 1 and map 2, some patients reported detecting and discriminating the sound, but they did not recognize it (they did not know what they were hearing). They detected but made mismatches between Ling sounds, between the instruments, and named instruments, such as *hiss* , *beat* , *a thin sound* , or *papapa* . With map 1, the rattle (that was not presented) was also mentioned, and one patient reported hearing well and one reported hearing it low. With map 2, three patients reported being too loud, one patient reported discriminating between low and high sounds. All these responses were considered detection. Only those who recognized the sound being presented were considered recognition. Results were similar for all patients in both maps, although discomfort was reported with map 2 (stronger) by 3 patients.

Regarding tNRT, Table 1 presents the descriptive statistics of tNRT in the OR and on activation day, and the mean difference between tNRT in the two situations.

**Table 1:** Comparison between tNRT values: in the operating room and at the activation day (after 30 days)

Electrode	Variable	Mean ± SD	Median (min–max)	Mean reduction (OR – activation)	*p* *
Electrode 1	tNRT OR	180.7 ± 19.6	182.5 (145–205)	14.8	< 0.001
	tNRT activation	165.8 ± 21.6	169.5 (126–191)		
Electrode 6	tNRT OR	187.3 ± 14.9	184.5 (162–215)	15.2	< 0.001
	tNRT activation	172.1 ± 10.8	173 (155–194)		
Electrode 11	tNRT OR	193.3 ± 16.5	199,5 (165–217)	13.1	0.015
	tNRT activation	180.3 ± 20.7	189 (134–203)		
Electrode 16	tNRT OR	184.1 ± 20.4	185 (141–220)	11.2	0.016
	tNRT activation	172.9 ± 16.4	176 (135–191)		
Electrode 22	tNRT OR	170.9 ± 19.2	175 (140–204)	13.8	0.091
( *n* = 11)	tNRT activation	157.1 ± 18.6	154 (129–190)		

OR = operating room, activation = activation day; min–max = minimum and maximum values; SD = standard deviation.

* Student's *t* -test for paired samples, *p* < 0.05.

Based on Table 1 , we can see that there was a statistically significant difference between the tNRT obtained in the OR and on the day of definitive activation for electrodes 1, 6, 11, and 16, but not for electrode 22 (however, *p* -value suggests that there is a tendency for a statistically significant difference).

Table 2 shows the descriptive statistics of tNRT in the OR and the measurements of C level informed by the patient on the day of activation and the mean difference between them. There was a statistical difference between the tNRT measurement performed in the OR and the measurement of the C level informed by the patient on the day of activation as a C level. We can observe that the values of the average and median of the current levels for each electrode decrease between one situation and another.

**Table 2:** Comparison between tNRT in the operating room and the C level assessment (activation with responses) (after 30 days)

Electrode	Variable	Mean ± SD	Median (min–max)	Mean reduction (OR –C)	*p* *
Electrode 1	tNRT OR	180.7 ± 19.6	182.5 (145–205)	40.7	< 0.001
	C level activation	139.9 ± 21.1	140 (102–175)		
Electrode 6	tNRT OR	187.3 ± 14.9	184.5 (162–215)	43.5	< 0.001
	C level activation	143.8 ± 14.1	146 (122–162)		
Electrode 11	tNRT OR	193.3 ± 16.5	199.5 (165–217)	46.4	< 0.001
	C level activation	146.9 ± 12.7	147.5 (130–165)		
Electrode 16	tNRT OR	184.1 ± 20.4	185 (141–220)	38.2	< 0.001
	C level activation	145.8 ± 12.3	147 (126–165)		
Electrode 22	tNRT OR	170.9 ± 19.2	175 (140–204)	29.4	0.001
( *n* = 11)	C level activation	141.5 ± 11.4	138 (127–165)		

Abbreviations: Activation, activation day; min–max, minimum and maximum values; OR, operating room; SD, standard deviation.

* Student *t* -test for paired samples, *p* < 0.05.

We can see clearly how in the three situations (tNRT on the day of surgery, tNRT on the day of definitive activation, and C level on the day of activation), the current level decreases.

Table 3 presents the descriptive statistics of IT in the OR and on the day of activation, 30 days later, and the average difference between IT in the two situations. There was a statistically significant difference between the two moments. It is clear how impedance values increased consecutively.

**Table 3:** Comparison between electrode Impedance telemetry (IT) in the operating room and in the activation day (after 30 days)

Electrode	Variable	Mean ± SD	Median (min–max)	Mean increase (activation – OR)	*p* *
Electrode 1	IT OR	57 ± 1.5	5.7 (3.9–8.7)	8.3	< 0.001
	IT activation	13.9 ± 2.2	14 (9.6–17)		
Electrode 6	IT OR	5.1 ± 2.1	5.1 (2.5–9.7)	7.9	< 0.001
	IT activation	13 ± 2.2	13.1 (7.7–15.7)		
Electrode 11	IT OR	5.6 ± 2.2	5.2 (2.9–10.9)	6.2	< 0.001
	IT activation	11.8 ± 2	12.2 (7.6–13.8)		
Electrode 16	IT OR	5.8 ± 2.0	5 (3.6–10.1)	5.6	< 0.001
	IT activation	11.4 ± 2.8	12.3 (6.6–15.1)		
Electrode 22	IT OR	6.3 ± 2.6	5.3 (3.2–10.6)	5.4	< 0,001
	IT activation	11.8 ± 3.3	12 (4.4–16.8)		

Abbreviations: activation, activation day; min–max, minimum and maximum values; OR, operating room; SD, standard deviation.

* Student *t* -test for paired samples, *p* < 0.05.

## Discussion

We observed that in our CI patients, it was possible to perform CI activation immediately after surgery, while still in the operating room (OR), and to use the tNRT as a C level of stimulation. There was a statistically significant difference in telemetry (both IT and tNRT comparing OR and day of activation), in that IT increased after surgery, and tNRT and C level decreased after surgery; in the OR, there was detection in behavioral tests with Ling sounds, musical instruments, and clapping, but discomfort to the sound was also reported. We suggest, therefore, using a lower current level for activation than the one found on tNRT on the day of surgery.

*Mappings* for CI take time and must be performed regularly, as the use of the processor itself requires new adjustments and personalized programming for each individual, aiming at better hearing gain with increasingly clear, crisp, and comfortable sound.

Objective tests are used in patients who cannot cooperate to identify the audible threshold and C level thresholds (T and C levels, respectively), so ECAP thresholds are used to aid programming. 3 Botros & Psarros (2010) 4 noted that, currently, the main clinical applications of NRT are to confirm correct implant function and lead introduction by obtaining ECAP, generally close to threshold, to control implant function over time, and to assist the adaptation and programming process, using ECAP thresholds as an audible level estimate. There are moderate correlations in T and C levels between psychophysical loudness assessments (behavioral levels) and those predicted by the ECAP. Although more objective tests currently exist, subjective procedures cannot be excluded or replaced entirely.

Every behavioral assessment needs instruments of low, medium, and high timbres and with weak, medium, and strong intensity, these instruments are: bell rattle, agogô, bell, drum, coconut shells, rattle, castanets, ganzá, reco-reco, caxixi, xylophone, triangle, black black, accordion, whistle, cymbals. There are also methods that are used in evaluation, such as knocking on the door and clapping hands. 5 Composing the behavioral assessment, Ling sounds, proposed by Daniel Ling, incorporate phonemes of low, medium, and high frequencies, which typically occur in speech. 6 The concept behind Daniel Ling's Six Sound Test was to select familiar speech sounds that broadly represent the 250-to-8,000 Hz speech spectrum. These methods are useful to address detection, discrimination, and identification skills, but they are not comprehension tests. 7 Auditory skills are: detection , the most basic level of sound perception, is awareness of the presence or absence of sound; discrimination, the ability to differentiate two or more stimuli, saying whether they are the same or not; recognition, skill that makes it possible to identify, classify, and name what you have heard; and listening comprehension , the most complex, which allows the individual to understand the meaning of language in oral speech. Attention and memory processes permeate these skills and are essential for their development. 7 8 9

Some tests are habitually performed, either intraoperatively or shortly after surgery while still in the OR, such as IT, which aims to assess the integrity and functionality of the electrode array, and NRT, which allows recording of the ECAP of the distal portion of the auditory nerve in patients using the implant itself to elicit the stimulus and record the ECAP responses, evaluating the functional characteristics of ganglion cells and other neural structures, 10 11 which may be useful for programming the speech processor during the first postoperative adjustment. 12 Neural Response Telemetry is obtained in ∼ 80% of the evaluated individuals, and its technique can be a valuable tool in confirming the integrity of the internal device, in the objective determination of which electrodes can be included in a given map, the best stimulation speeds and speech coding strategies, as well as the estimation of T and C levels, with extreme clinical importance. 13 Electric compound action potential thresholds can be useful to predict the minimum and maximum levels that determine the dynamic area for electrical stimulation; these levels can be named and defined differently for the different brands of CI on the market. The dynamic area is the region between the amount of current that first induces the auditory sensation, that is, the threshold for electrical stimulation (T level) and the maximum intensity sensation level that the patient will accept for electrical stimulation (C level). This is done so that the CI is programmed within the loudness range which allows speech sounds and other sounds to be audible but not uncomfortable. 14 The dynamic area is usually determined through psychophysical measurements, although objective or electrophysiological measurements of hearing can be used. 15 However, the correlation between ECAP thresholds and psychophysical thresholds is affected by many factors. 3 With the use of local anesthesia and sedation, 2 at the end of the surgery, still in the OR, the patients are already awake and when the NRT is performed, they report listening to the stimuli that occur during the testing and sometimes questioning if that is what they will hear afterwards. With this report, we realized that it would be possible, even in the OR, to activate the speech processor, allowing the patient to have auditory perception immediately after the surgery.

Lai et al. (2004) 16 showed that intraoperative NRT data were generally stable enough to be used to assist in initial speech processor mappings, and it was not possible to predict changes in the map's subjective threshold or comfortable loudness levels based on changes observed in the NRT data. In our study, we warn that this should be done with caution, because there was a statistically significant difference when comparing tNRT responses on the day of surgery with the measurement of C level (comfort threshold) on the day of activation. These values decreased, tNRT in the OR was higher than on the day of definitive activation, and this, in turn, was higher than the C level measured on the day of definitive activation. Unlike tNRT, we observed that impedance values increased from the day of surgery to the day of activation, but it should be noted that IT was the first procedure performed, and the stimulation current had still passed. Often, when we perform CI activation in young children who do not cooperate or who do not allow tNRT to be performed, we use tNRT performed in the OR as basis for building the activation map. It is important to know, although it is information given by an adult, how much the tNRT data performed on the day of surgery can help but also be uncomfortable in the listening experience. In this study, we observed that the map with the C level at the tNRT threshold, although considered uncomfortable by two patients, because it was higher, allowed the detection of Ling sounds, 6 clapping, and the detection of instruments, with the patients only reporting whether they were bass or treble. Of course, with children we should rely much more on observing behavioral responses and make use of other objective measures such as the investigation of the electrically-evoked stapedial reflex threshold. 17 It is important to emphasize that when programming levels are determined based on ECAP thresholds, the stimulation can be uncomfortably high, particularly in the basal electrodes. 18 19 20 We could observe that there was a decrease of ∼40 cu for the basal electrodes between the ECAP thresholds (tNRT) on the day of surgery (OR) and the C level reported by the patient on the activation day.

Behavioral measures, even if minimally observable, are important for CI programming. Objective electrophysiological measurements help predict behavioral levels, but these alone cannot replace the accuracy of a behavioral map. 21 Research has revealed a stronger correlation between ECAP threshold and C level than between ECAP threshold and T level. 22 We believe that our patients found it easy to detect the sound and even recognize it, because they had all postlingual hearing loss and already wore a CI in one ear or hearing aids. It was possible to observe with map 2, level C at the NRT threshold, a higher percentage of detection and recognition of Ling sounds 6 when compared with map 1. Regarding the instruments, we used instruments with different sound spectrum. For example, for the bell rattle, detection was considered the fact that they identified the sound and reported it being strong or weak and high or low. We could observe that only for one ear with map 2 the bell rattle was not detected. In this research, it was possible to observe that immediately after the insertion of the electrode bundle in the cochlea, while still in the OR, the patient can already present auditory detection and recognition responses, and this auditory experience makes them calmer to wait for the definitive activation date in 30 days.

There may be some possible limitations to this study, such as a small sample, which could prevent results from being generalized. We also did not include patients with prelingual deafness or children, because these patients had no prior hearing experience.

We believe in the importance of activation as early as possible, but we agree that the healing period must be respected.

## Conclusion

This study shows that CI activation in the OR, immediately after surgery with local anesthesia and sedation, is possible. We could provide the patient with a hearing experience with the CI while still in the OR with auditory detection and auditory discrimination of different types of stimuli, but with poor recognition. Maps with higher current offer better results, but also provide auditory discomfort. Impedance telemetry values increased from the day of surgery to the day of definitive activation, at 30 days, and NRT values decreased for the same period, and both were statistically significant.
